# A discussion of the development of an instrument to measure anorexic symptomatology in men

**DOI:** 10.1186/2050-2974-3-S1-O52

**Published:** 2015-11-23

**Authors:** Megan Reeves

**Affiliations:** 1 University of the Witwatersrand, Johannesburg, South Africa

## 

Quantitative studies comparing the incidence of AN symptomatology in men and women often utilise measures that were developed for use amongst female samples. The findings of these studies may lead to a misrepresentation of the clinical picture of AN in men since these instruments carry potential gender biases and thus often yield skewed results. Therefore, the current prevalence statistics of anorexic symptomatology amongst men that may be found in the literature may be inaccurate. The development of a screening tool specifically designed to assess levels of anorexic symptomatology in men would lead to a more accurate measurement of mens' experiences of AN. This paper will outline the development of such a measure based on an integrative review of the literature over the period between July 2000 and July 2013 and using Google Scholar and South African e-Publications. Data from a thematic analysis of interviews with clinical psychologists and psychiatrists in South Africa was also used in the development of the instrument. The instrument is presented with a view to discussing the way forward for the development of a more accurate understanding of the levels of AN symptomatology in the male population.

